# Unmet Needs in the Management of Chronic Kidney Disease-Associated Pruritus and the Characteristics of the Ideal Treatment: A Spanish Cross-Sectional Survey from a Multidisciplinary Perspective

**DOI:** 10.3390/jcm14020624

**Published:** 2025-01-19

**Authors:** Patricia De Sequera, José M. Martínez-Sesmero, Isabel Romo, Ana Calvo, Susana Aceituno, Olga Ruiz-Andrés, Juan C. Julián-Mauro

**Affiliations:** 1 Nephrology Department, University Hospital Infanta Leonor, 28031 Madrid, Spain; 2 Deputy Medical Director, Lozano Blesa Clinical Hospital, 50009 Zaragoza, Spain; 3 Head of the Quality, Innovation and Health Outcomes Unit, OSI Barrualde Galdakao, Osakidetza, 48960 Vizcaya, Spain; 4 Health Economics & Outcomes Research, Outcomes’10 (a ProductLife Group Company), 12071 Castellón, Spain; 5 Value Access Policy Manager, CSL Vifor, 08028 Barcelona, Spain; 6 Management, Association for the Fight against Kidney Diseases ALCER, 28002 Madrid, Spain

**Keywords:** CKD, pruritus, CKD-aP, BWS, preferences, attributes, treatment, patient management, QoL

## Abstract

**Objectives:** Chronic kidney disease-associated pruritus (CKD-aP) is underdiagnosed and not fully understood by healthcare professionals, which leads to poor patient management and impacts patients’ quality of life (QoL). The aim of this study was to analyse unmet needs in CKD-aP management and explore the attributes/characteristics that the ideal CKD-aP treatment should have from the perspective of a group of nephrologists, hospital pharmacists, nurses, patient representatives, and regional health authorities in Spain. **Methods:** A descriptive, cross-sectional study was conducted using an e-survey including ad hoc questions (6-point Likert scale) related to unmet needs in CKD-aP and best–worst scaling (BWS) to prioritise the attributes/characteristics of the ideal CKD-aP treatment. The survey was developed from a literature review, a patient focus group, and a multidisciplinary expert committee. **Results:** A total of 21 people participated, and it was considered, among other aspects, that CKD-aP had a significant impact on patient QoL (4.29/5), but the diagnosis rate and knowledge level of agents involved, as well as current treatment efficacy and safety, were low (1.71/5, 2.19/5, 1.91/5, and 2.67/5, respectively). The attributes “improves overall QoL (physical and mental)”, “reduces itch with statistical significance”, and “treatment is supported by clinical development/high evidence and has AEMPS (Spanish Agency for Medicines and Medical Devices)-approved indication for pruritus” were selected as the most valued attributes. There was a positive balance between best–worst scores (86-5, 71-2, and 78-13 points, respectively). **Conclusions:** The results show the need to undertake actions to drive relevant changes in current clinical practice to improve CKD-aP diagnosis and management.

## 1. Introduction

Chronic kidney disease (CKD) is a complex condition that affects approximately 850 million people worldwide, over 85 million in Europe, and more than 6 million in Spain [1]. As CKD progresses, complications such as mineral bone disorders, anaemia, hypertension, and hypokalaemia may develop, leading to advanced stages of CKD, and patients may require renal replacement therapy and haemodialysis [2]. Patients undergoing haemodialysis report a high symptom burden, including fatigue, pain, mood disturbance, sleep disturbances, and pruritus [3]. Chronic kidney disease-associated pruritus (CKD-aP) is one of the most common and disabling comorbidities in patients with advanced CKD [4], affecting up to 80% of patients on haemodialysis (HD) [5]. Pruritus is defined as the sensation of itching in one area or all over the body that causes the need to scratch [6]. It is an extremely distressing and debilitating symptom comparable to chronic pain [7]. The severity varies from somewhat uncomfortable to extremely disturbing and inducing restlessness [8]. CKD-aP in these patients has a significant impact on their quality of life (QoL), leading to sleep disruption and mental disorders [9]. Pruritus is associated with an increased risk of dementia, cardiovascular disease, and mortality [10,11,12,13,14,15,16]. In addition, there is a statistically significant association between increased itchiness severity and worsening QoL [17,18].

It is an old and well-known health problem, the prevalence of which has decreased with the improvement of dialytic techniques, but it still persists [6]. In Spain, according to the National Registry of Renal Patients, the prevalence of patients with HD was 26,683 in 2021 [19]. A survey (*n* = 1605) conducted by the Spanish Society of Nephrology (SEN) in 2021, mostly on HD (92%), reported that 50.5% of patients suffered from pruritus and that 27% of the total suffered from moderate to severe pruritus [20]. An international cohort study of adult dialysis patients (Dialysis Outcomes and Practice Patterns Study (DOPPS)) reported similar figures: 59% of Spanish patients suffered from pruritus and 36% had moderate to severe pruritus [21]. However, despite having CKD-aP, 35% of HD patients stated that they never reported their pruritus symptoms to a healthcare professional [22], and only 16% of nephrologists in Spain indicated that the prevalence of pruritus in their centres was greater than 20%, which shows a clear inconsistency between prevalence and diagnosis [6]. Moreover, the use of scales to measure it and its codification in medical records is not well established. The diagnosis of CKD-aP is mainly based on direct communication of the patient’s symptoms to healthcare staff [6].

Recently, in Spain, a consensus document with recommendations for the diagnosis and management of CKD-aP in HD patients was published [4]. This document, which was subject to public consultation by all members of the Spanish Society of Nephrology (SEN) prior to publication, addresses some of the identified barriers to adequately managing this condition. These included which scales are more appropriate for CKD-aP diagnosis and recommendations for the codification of the disease in patient medical records with the tools available today.

The multifactorial pathogenesis of CKD-aP is still not fully understood, and different explanations have been considered possible [23,24]. Various pruritogens, receptors, neurons, and neurotransmitters have been identified as playing a role in the pathophysiology of itching [25]. It is likely that different itch syndromes involve distinct combinations of cells and molecules responsible for transmitting itch sensations [25]. While few studies have focused on the specific pathophysiological mechanisms of itch in CKD-aP, general theories have emerged from the existing literature [25]. CKD-aP has been associated with dermatological factors (xerosis or skin barrier dysfunction), systemic factors such as immune system dysfunction and the proinflammatory state inherent to CKD, neurological factors, and factors due to the accumulation of toxins and other metabolic substances, including parathyroid hormone, calcium, phosphorus, and aluminium [25]. The fact that healthcare professionals do not fully understand CKD-aP, together with the fact that it is underreported by patients, leads to underdiagnosis, and it usually goes unnoticed by most nephrologists [6,9].

Besides the challenges around diagnosis, there are also difficulties around the management of this disease, and there is not a commonly accepted clinical practice guideline. As there is currently no approved treatment for CKD-aP available in Spain [26], off-label pharmacological treatment approaches are used to tackle CKD-aP, such as antihistamines, pregabalin, and antidepressants. However, the evidence on the efficacy of these therapies in CKD-aP is limited [6,9]. In the limited available clinical evidence, some of these therapeutic approaches have shown good results in reducing itchiness, but they have many serious side effects, which make them inappropriate for use in most HD patients, as they are frail patients [6,9,27,28,29,30,31,32,33,34,35,36]. Furthermore, these are polymedicated patients, so it is a priority to treat pruritus with minimal pharmacological burden and safe drugs while avoiding drug–drug interactions as much as possible [9]. Therefore, patients experiencing CKD-aP face clear unmet medical needs, with studies reporting high numbers of untreated patients or, when under treatment, high patient dissatisfaction [37].

Understanding unmet needs, the impact on QoL, and patients’ and healthcare professionals’ perspectives about CKD-aP may help in clinical, licensing, reimbursement, and policy decisions. Therefore, the objective of this study was to analyse current unmet needs in CKD-aP management and preferences from a multi-stakeholder perspective regarding the attributes that the ideal CKD-aP treatment should have.

## 2. Materials and Methods

This is a descriptive, cross-sectional study based on an electronic questionnaire, including ad hoc questions related to unmet needs in CKD-aP, and a best–worst scaling (BWS) experiment.

### 2.1. Participants

The study sample included health professionals (nephrologists, hospital pharmacists, and nurses) who are experts in CKD management but have different levels of expertise in the disease, as well as representatives of patient advocacy groups and regional health authorities with decision-making responsibilities. The participants were identified and invited by the ALCER (Spanish Association for the Fight against Kidney Diseases). Fifty-three people received the invitation to participate in the project with the questionnaire link, username, and password (exclusive for each participant) via e-mail (between May 2023 and September 2023).

A multidisciplinary committee of experts (n = 4) consisting of one nephrologist expert in CKD-aP, one hospital pharmacist, one patient representative from ALCER, and one regional health authority participated in the design of the questionnaire and the interpretation of results.

### 2.2. Ethical Considerations

All procedures followed were in line with the ethical standards of the Declaration of Helsinki. Due to the nature of the study, which did not collect clinical data from participants, including data on drugs or interventions, and the fact that the questions were related to participants’ perceptions, the approval of an ethics committee was not required, as defined in the Royal Decree 957/2020 of 3 November [38] and the Memorandum of Cooperation between Ethics Committees [39]. Prior to beginning the questionnaire, all participants were informed of the study structure, scientific committee, and the confidentiality and data protection measures in place. Written informed consent was obtained by all participants by checking a box indicating that they read and approved the survey and agreed to participate in the study with all the conditions described.

### 2.3. Survey

The survey’s content was developed based on a literature review dealing with studies on the management of CKD-aP (Supplementary Methods and Table S1), information from a focus group with patients, and the opinion of the scientific committee. There was no developed survey to assess unmet needs in CKD-aP. Therefore, it was considered appropriate to conduct a literature review to identify those relevant aspects of the diagnosis and management of the disease where gaps exist in order to develop questions to explore the perspectives of different stakeholders.

The online patient focus group included five people who suffered or had suffered with CKD-aP—two with mild–moderate pruritus and three with moderate–severe pruritus—while on renal replacement therapy, and was conducted to identify their needs and demands about disease management. Patients were presented with the information identified in the literature. Different questions were asked for the discussion: the symptoms of the pathology, diagnosis, the impact of the disease and its treatment on their daily life, and their perception of its management during medical visits. Patients were contacted and invited to participate via the ALCER.

Finally, an online meeting was held with the multidisciplinary committee to review the information gathered from the literature and the focus group and to design the questionnaire (for the full questionnaire, see Table S2).

The first part of the questionnaire consisted of participant baseline characteristics (age, sex, autonomous region, profile). In the second part, unmet needs about CKD-aP pathology management were identified using a 6-point Likert scale for each item (Table 1). Depending on the question included in the topic, the unmet need is greater when the score is closer to 5 or 0.

In the third part, we conducted a BWS exercise to elicit participants’ preferences about the most and least preferred attributes that an ideal treatment for CKD-aP should have. Our objective in the BWS analysis was to establish a ranking from the response counts, not to make inferences and comparisons from the sample of participants; so, considerations that might imply a more accurate sample size estimate were not required [40]. Once the attributes that would form part of the BWS were defined by the scientific committee (in this case, characteristics that would make an ideal treatment for CKD-aP), they were combined to form the choice scenarios using a balanced incomplete block design [41]. This design ensures that each attribute appears the same number of times within the design and that all pairs of attributes are combined together the same number of times [40,41]. The attributes were identified during the literature review, and the patient focus group validated the relevance of potential attributes from the Spanish patients´ perspectives. The attributes were also verified by the experts committee for inclusion in the BWS (Table 2).

Each scenario comprised four attributes, each attribute appeared in seven scenarios, and each pair of attributes appeared together three times.

### 2.4. Statistical Analysis

Stata version 14 was used for the statistical analysis. The participants’ characteristics were summarised through descriptive statistics (continuous variables were presented as means and standard deviations (SDs) and categorical variables as relative and absolute frequencies). The results from the Likert scale were presented using minimum and maximum values for continuous variables in addition to the mentioned statistics. For the BWS, a best-minus-worst score was calculated by subtracting the number of times an attribute was chosen as worst from the number of times it was chosen as best. Subgroup descriptive analyses were performed for each group of participants.

## 3. Results

### 3.1. Participants

The questionnaire was ultimately completed by 21 participants (a 40% response rate) from 11 regions of Spain: five nephrologists, five hospital pharmacists, three nurses, four patients (one patient advocacy group representative and three expert renal patients), and four health authorities. The mean age of all participants was 53 years old, and 57% were men (for all participants’ sociodemographic data, see Table S3).

### 3.2. Unmet Needs in CKD-aP Management

**Figure 1:**
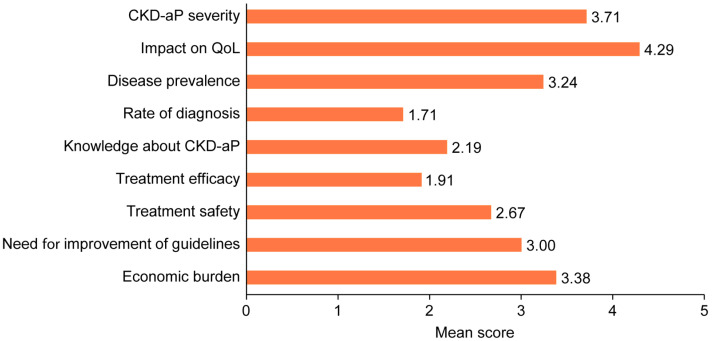
Unmet needs identified by participants. CKD-aP: chronic kidney disease-associated pruritus; QoL: quality of life.

Participants considered CKD-aP as a moderate to severe pathology (3.71/5) and that it had a very severe impact on patients’ QoL (4.29/5). In addition, they reported that the size of the population affected by pruritus is moderately high (3.24/5). However, the diagnosis rate was low (1.71/5). Otherwise, they identified that it was necessary to improve levels of knowledge (2.19/5) and the guidelines and consensus documents for CKD-aP management (3.0/5). In addition, the low–moderate score given for the efficacy and safety of current treatments (1.91 and 2.67, respectively) showed unmet needs in these areas. Furthermore, participants considered that the economic burden (3.38/5) of CKD-aP management was moderately high (Figure 1).

**Figure 2:**
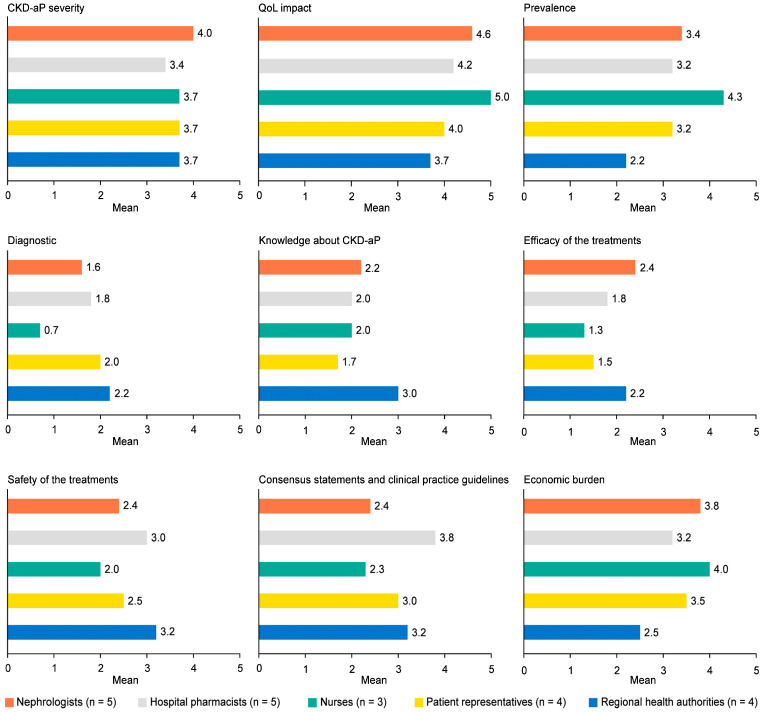
Unmet needs identified by subgroups. CKD-aP: chronic kidney disease-associated pruritus; QoL: quality of life.

Nurses and nephrologists were the highest scoring profiles, and the regional health authorities’ profile was the lowest (Figure 2). The item “CKD-aP severity” obtained similar scores across all profiles but not the rest of the items (for the full unmet needs identified by profile, see Table S4). Regarding the “impact on patient QoL”, nurses and nephrologists stood out, although all scored close to 4. Concerning “disease prevalence”, nurses’ opinions were the most prominent (4.3/5) and regional health authorities were the least (2.2/5). Regarding “diagnosis rate”, all profiles provided low scores (maximum 2.2). The only item in which patients scored lower than the rest of the profiles was “knowledge about CKD-aP”. In this case, the health authorities’ group score was the highest. Concerning the efficacy of current treatments, low–medium scores were obtained, from 1.3 for nurses and 1.5 for patients to 2.4 for nephrologists. Regarding the safety of current treatments, its scores were better than for efficacy, but nurses and patients still scored lower than the others. The greatest variability in responses was in the item for the “need to improve guidelines and consensus documents” (details about variability are included in Table S5). In this case, hospital pharmacists indicated the highest score (3.8/5), followed by regional health authorities (3.2/5) and patients (3.0/5). Regarding the cost of disease management, all profiles, except for regional health authorities (2.5/5), obtained similar scores (between 3.2 and 4.0).

### 3.3. Attributes of Ideal CKD-aP Treatment by BWS

The preferences of respondents for each attribute were indicated by the distance between the least and most preferred levels (Table 3). According to the best-minus-worst score, the highest valued attribute level was “treatment improves the overall QoL (physical and mental)” and the lowest was “the treatment is administered in the dialysis circuit after each dialysis session”.

**Figure 3:**
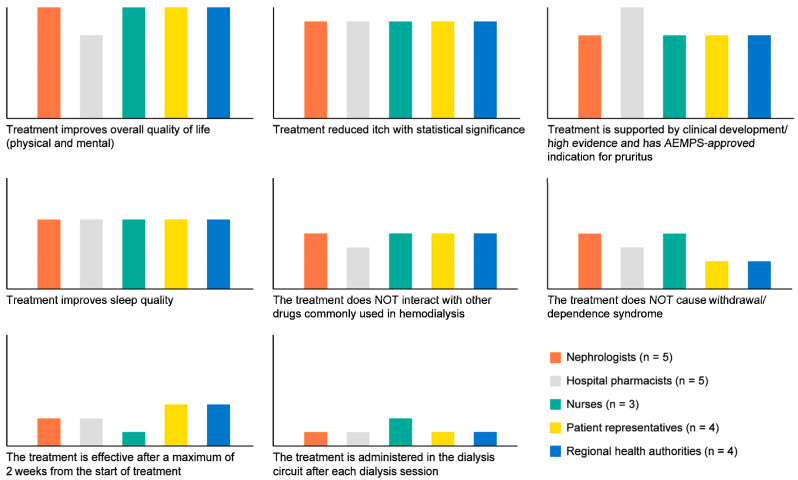
Preferences about which attributes the ideal CKD-aP treatment should have by subgroups. AEMPS: Spanish Agency of Medicines and Medical Devices.

The three best attributes occupied the first three positions in all groups (Figure 3). However, hospital pharmacists prioritised “treatment is supported by clinical development/high evidence and has Spanish Agency of Medicines and Medical Devices-approved indication for pruritus”.

All groups ranked “treatment improves sleep quality” in the fourth position. Hospital pharmacists prioritised that the treatment did not cause withdrawal/dependence syndrome, and the remaining groups did not. Similarly, the nurse profile gave more importance to administering the drug than the other groups. Patient representatives prioritised “the treatment is effective after a maximum of 2 weeks from the start of treatment” over “the treatment does not cause withdrawal/dependence syndrome” and “the treatment is administered in the dialysis circuit after each dialysis session” (detailed results are included in Table S6 in Supplementary Materials).

## 4. Discussion

Pruritus is a common and burdensome issue reported by the general population, but its prevalence and severity are even higher in patients with CKD [42]. Furthermore, these individuals have an increased risk of developing skin ulcers that are difficult to treat (particularly in those with diabetes or arteriosclerosis). Thus, it would be interesting to include patients with pruritus in preventive treatment protocols for skin lesions [43,44,45]. In recent years, there has been growing national and international interest in gaining a better understanding of CKD-aP prevalence, symptoms, and treatment, especially its impact on patients’ health-related QoL [6,7,9,46,47]. This study reported the unmet needs for CKD-aP management and the preferences for the characteristics that a hypothetical, ideal CKD-aP treatment should have from the perspective of a group of nephrologists, hospital pharmacists, nurses, patient representatives, and regional health authorities. To our knowledge, this is the first such study to survey all stakeholders involved in disease management nationwide in Spain.

Our study finds that CKD-aP is considered a serious pathology that greatly impacts QoL in dialysis patients. This is consistent with numerous national and international studies on this topic [7,14,18,20,21,22,46,48,49,50,51,52]. In addition, the size of the population affected by pruritus is quite high; however, the low diagnosis rate suggests there may be a larger affected population than currently identified. This underdiagnosis could be due to the identified need to improve knowledge about CKD-aP and better information in guidelines and consensus documents. Other recent studies carried out in Spain [6,47] and other European countries [7] have shown results that are in line with those highlighted in our study. Accordingly, in a survey conducted by the European Kidney Patients Federation (EKPF), 75% of nephrologists interviewed agreed that CKD-aP is underdiagnosed in HD patients and emphasised the lack of guidelines and standardised scales to consistently diagnose and classify CKD-aP severity. In this context, the recently published consensus document [4] in Spain provides national recommendations for the diagnostic and therapeutic management of CKD-aP, aiming to address the current lack of specific guidelines for its diagnosis and management.

Besides the challenges around diagnosis, participants also identified difficulties around the medical needs of this disease. Currently, in Spain, there is no approved, available therapeutic option to tackle CKD-aP [26]. Furthermore, CKD-aP codification in medical records is not usual, as there are no specific codes for the disease in the ICD-9 or ICD-10 (Statistical Classification of Diseases and Related Health Problems in Spain). Previous work [53] has confirmed that the lack of specific codes reduces diagnosis by experts, and the results of this study would be in line with these findings. In addition, they pointed out that the current economic burden of CKD-aP management in dialysis patients is high.

Regarding the subgroup analysis and based on the opinion of the expert committee of this study, it was identified that nurses had the highest scores, likely because of their tremendous involvement in the daily care of dialysis patients. On the contrary, regional health authorities reported the lowest unmet needs, which aligns with their expected roles and responsibilities. In particular, regional health authorities highlighted items such as the size of the affected population, the level of knowledge that the agents involved in the pathology have, and the costs associated with managing patients with CKD-aP. On the other hand, it was expected that patient representatives would have higher scores than nurses; nevertheless, patients showed their objectivity as experts when assessing CKD-aP as one symptom among the diverse array of end-stage CKD symptoms in dialysis treatment as a whole. Hospital pharmacists showed a mid-level score profile and highlighted the need to improve guidelines and consensus documents, as they play an important part in their responsibilities.

CKD-aP severity and its impact on QoL are the items with the lowest variability in response across profiles (minimum: 3, maximum: 5.0, and SD: 0.64 and 0.56, respectively). The need to improve guidelines and consensus documents is the item with the greatest variability (minimum: 1.0, maximum: 5.0, and SD: 1.18). In this case, hospital pharmacists indicated the highest score, even higher than nephrologists or nurses, which may be because the latter two are regularly updated with scientific information and believe that they have adequate information for the proper management of these patients.

According to the best-minus-worst score, three of the four most valued attributes by the total sample were related to patient-reported outcomes (PROs): “treatment improves the overall QoL (physical and mental)”, “treatment reduces itch with statistical significance”, and “treatment improves sleep quality”. All groups also considered “treatment is supported by clinical development/high evidence and has Spanish Agency of Medicines and Medical Devices-approved indication for pruritus” as one of the most relevant attributes, being prioritised by hospital pharmacists. The remaining attributes varied in order, depending on the interests and responsibilities of each group. Therefore, pharmacists’ prioritisation could be due to their responsibilities to ensure effective, safe, and efficient treatment, while patients prioritised attributes with the aim of alleviating symptoms as soon as possible. Finally, it is noteworthy that patient representatives and regional health authorities had similar attribute prioritisation, reflecting the results of the efforts made in recent years to promote patient-centred care, now considered an essential goal of high-quality healthcare systems.

## 5. Limitations

This study had some limitations. First, the sample size was too small to be regarded as representative of the country. However, a balanced sample was included in each group of participants, ensuring that key multi-stakeholder perspectives were considered in analysing the different criteria for disease management. The sample was distributed among eleven different regions within the territory, which ensured broad representation despite the sample size. Notably, given the lack of formal guidance on the minimal sample size required for best–worst scaling [40,54] and considering that making inferences about the population represented via the sample or comparing scores between groups was not the objective of this study, we aimed to reach as many participants as possible. However, the response rate ultimately determined the sample proportion. The number of participants and analyses is in line with other studies featuring similar methodologies in the recent literature [55,56,57]. Secondly, participants’ preferences and choices are constrained within the attributes presented in BWS scenarios; thus, societal preferences for the ideal treatment of CKD-aP may include attributes not explored in this study. Nonetheless, the attributes tested herein were selected according to the literature and the opinions of a multidisciplinary committee of experts. The BWS method is becoming increasingly popular in eliciting preferences in healthcare [58] because it helps collect more data compared to discrete choice experiments and has several advantages over these [59]. On the one hand, respondents are provided with scenarios one by one rather than two (or more) at a time; thus, BWS is considered less cognitively demanding for participants. On the other hand, respondents make choices within scenarios rather than between scenarios.

Despite these limitations, this study provides valuable insights into current CKD-aP management, an underdiagnosed comorbidity that significantly impacts patients’ quality of life and presents unmet needs recognised by different stakeholders involved in its management. Beyond raising awareness of the relevance of CKD-aP, this study serves as a starting point for establishing measures from both clinical and health policymaker perspectives to address the identified needs related to diagnosis, management, and treatment availability for this population. Future efforts should focus on identifying and diagnosing this pathology, measuring its severity and impact on patients’ lives, and optimising treatment, favouring the use of safe and effective treatments for these patients. Pruritus should always be considered a key component in any preventive treatment protocols for skin lesions.

## 6. Conclusions

Based on the perspectives of a multidisciplinary panel of experts, this study has established the unmet needs and preferred attributes of an ideal treatment for CKD-aP management in Spain. The results suggest that CKD-aP is a severe pathology with a high impact on QoL in dialysis patients. Moreover, it is underdiagnosed or not fully understood by healthcare professionals. Thus, strengthening our understanding and knowledge of CKD-aP should be a priority.

This study concludes that the ideal CKD-aP treatment should improve overall QoL (physical and mental) and have an approved indication for CKD-aP supported by clinical development/high-quality evidence, according to participants’ perspectives.

This study serves as a starting point to raise awareness about CKD-aP—an enduring health problem that still persists in our society—and to create an action framework with all stakeholders involved in CKD-aP management to provide viable solutions for patients with this disease.

## Figures and Tables

**Table 1:** Items assessing CKD-aP pathology management.

Item 1: Degree of severity	Question: How severe do you consider the CKD-aP? Likert scale: 0 none → 5 high
Item 2: Impact on QoL	Question: How do you consider that CKD-aP affects the QoL of patients? Likert scale: 0 none → 5 high
Item 3: Size of the population affected	Question: How do you consider the size of the population affected by CKD-aP? Likert scale: 0 little affected population → 5 a lot affected population
Item 4: Rate of diagnosis	Question: Regarding the total number of patients suffering from CKD-aP, how do you consider the rate of diagnosis of CKD-aP? Likert scale: 0 low → 5 high
Item 5: Level of knowledge of the agents involved in the pathology management	Question: How do you consider the level of knowledge that the agents involved in the pathology have about CKD-aP? Likert scale: 0 low → 5 high
Item 6: Efficacy of treatments currently used	Question: Although there are no authorised drugs available in Spain for CKD-aP, how effective do you consider the treatments currently used to be? Likert scale: 0 little → 5 a lot
Item 7: Safety of treatments currently used	Question: Although there are no authorised drugs available in Spain for CKD-aP, how safe do you consider the treatments currently used to be? Likert scale: 0 little → 5 a lot
Item 8: Current patterns included in the guidelines and consensus document	Question: How do you consider the current patterns for the management of CKD-aP included in the guidelines and consensus documents? Likert scale: 0 not at all improvable → 5 very improvable
Item 9: Cost associated with the management of a patient with CKD-aP	Question: How do you consider the cost associated with the management of a patient with CKD-aP compared to one without pruritus? Likert scale: 0 low → 5 high

CKD-aP: chronic kidney disease-associated pruritus; QoL: quality of life.

**Table 2:** Attributes included in the BWS.

Attributes included in the BWS
The treatment is administered in the dialysis circuit after each dialysis session
The treatment is effective after a maximum of two weeks from the start of treatment
Treatment reduces itch with statistical significance
The treatment does NOT interact with other drugs commonly used in HD
The treatment does NOT cause withdrawal/dependence syndrome
Treatment improves overall QoL (physical and mental)
Treatment improves sleep quality
Treatment is supported by clinical development/significant evidence and has Spanish Agency of Medicines and Medical Devices (AEMPS)-approved indication for pruritus

HD: haemodialysis; QoL: quality of life.

**Table 3:** Participants’ preferences about which attributes the ideal CKD-aP treatment should have.

Attributes of Ideal CKD-aP Treatment by BWS	Best	Worst	B–W Score
Treatment improves overall QoL (physical and mental)	86	5	81
Treatment reduces itch with statistical significance	71	2	69
Treatment is supported by clinical development/significant evidence and has Spanish Agency of Medicines and Medical Devices-approved indication for pruritus	78	13	65
Treatment improves sleep quality	32	18	14
The treatment does NOT interact with other drugs commonly used in HD	14	25	−11
The treatment does NOT cause withdrawal/dependence syndrome	9	57	−48
The treatment is effective after a maximum of two weeks from the start of treatment	3	75	−72
The treatment is administered in the dialysis circuit after each dialysis session	1	99	−98

CKD-aP: chronic kidney disease-associated pruritus; QoL: quality of life; HD: haemodialysis. Higher values for the B–W score indicate that the attribute was chosen more frequently as most preferred, and negative values indicate that the attribute was chosen more frequently as least preferred.

## Data Availability

All data presented in this study are available on the supplementary.

## Supplementary Materials

The following supporting information can be downloaded at: https://www.mdpi.com/article/10.3390/jcm14020624/s1, Table S1: List of terms in the literature search strategy; Table S2: Online questionnaire; Table S3: Sociodemographic characteristics; Table S4: Unmet needs identified by profile; Table S5: Unmet needs. Variability in responses; Table S6: Preferences about which attributes the ideal CKD-aP treatment should have, detailed per group.
