# Supplementary material for: Unmet Needs in the Management of Chronic Kidney Disease-Associated Pruritus and the Characteristics of the Ideal Treatment: A Spanish Cross-Sectional Survey from a Multidisciplinary Perspective
Source: J Clin Med. 2025 Jan 19;14(2):624. doi: 10.3390/jcm14020624 (PMC11766129; doi:10.3390/jcm14020624)
Supplement: Supplementary file 1 [file jcm-14-00624-s001.zip › jcm-3367562-supplementary.pdf]

1.1. Table S1. List of terms in the literature search strategy

Methods

The PubMed search engine was used to gather the most recent information on CKD-aP management. A Boolean search (OR and AND) and MesH (Medical Subject Heading) terms of the PubMed data set were implemented to combine a range of keywords. Information was also obtained by manual searches, from Google/Google Scholar and national and international scientific societies such us ALCER, the Spanish Society of Nephrology, the European Dermatology Forum (EDF) or the European Academy of Dermatology and Venereology (EADV), among others.

Table S1

**Terms related to the pathology**	
1	Pruritus [MeSH]
2	Pruritus [tiab]
3	Chronic kidney disease [MeSH]
4	Chronic kidney disease [tiab]
**Terms related to unmet needs**	
5	Severity [tiab]
6	Quality of life [tiab]
7	Prevalence [tiab]
8	Incidence [tiab]
9	Diagnosis [tiab]
10	Knowledge [tiab]
11	Treatment [tiab]
12	Safety [tiab]
13	Guide [tiab]
14	Consensus [tiab]
15	Burden of disease [tiab]
16	Burden [tiab]
**Terms related to a national context**	
20	Spain [All fields]
21	Spanish [All fields]
(OR 1-2) AND (OR 3-4) AND (OR 5-16) // (OR 1-2) AND (OR 3-4) AND (OR 5-16) AND (OR 20-21). Search filters applied: the last 10 years. Priority was given Spanish and European studies with abstract available.	

CONFIDENCIAL

Confidencial No está permitida su distribución

1.2. Table S2. Online questionnaire

Part 1. Participants' sociodemographic characteristics	
**Age**	________years
**Sex**	☐ Male<br>☐ Female
**City or autonomous community where you work/live:**	☐ Andalucía<br>☐ Aragón<br>☐ Asturias<br>☐ Baleares<br>☐ Canarias<br>☐ Cantabria<br>☐ Castilla-La Mancha<br>☐ Castilla y León<br>☐ Cataluña<br>☐ Comunidad Valenciana<br>☐ Extremadura<br>☐ Galicia<br>☐ La Rioja<br>☐ Madrid<br>☐ Murcia<br>☐ Navarra<br>☐ País Vasco<br>☐ Ceuta y Melilla
**Profile**	☐ Nephrologists<br>☐ Hospital pharmacy<br>☐ Nurses<br>☐ Representatives of Patient Associations<br>☐ Health Authorities
**Part 2. Items about CKD-aP pathology management**	
**Item 1: Degree of severity**	Question: How severe do you consider the CKD-aP?<br>Likert scale: 0 none → 5 high

**Item 2: Degree of severity**	Question: How do you consider that CKD-aP affects the QoL of patients?<br>Likert scale: 0 none → 5 high
**Item 3: Size of the population affected**	Question: How do you consider the size of the population affected by CKD-aP?<br>Likert scale: 0 little affected population→ 5 a lot affected population
**Item 4: Rate of diagnosis**	Question: Regarding the total number of patients suffering from CKD-aP, how do you consider the rate of diagnosis of CKD-aP?<br>Likert scale: 0 low → 5 high
**Item 5: Level of knowledge of the agents involved in the pathology**	Question: How do you consider the level of knowledge that the agents involved in the pathology have about CKD-aP?<br>Likert scale: 0 low → 5 high
**Item 6: Efficacy of treatments currently used**	Question: Although there are no authorised drugs available in Spain for CKD-aP, how effective do you consider the treatments currently used to be?<br>Likert scale: 0 little → 5 a lot
**Item 7: Safety of treatments currently used**	Question: Although there are no authorised drugs available in Spain for CKD-aP, how safe do you consider the treatments currently used to be?<br>Likert scale: 0 little → 5 a lot
**Item 8: Current patterns included in the guidelines and consensus document**	Question: How do you consider the current patterns for the management of CKD-aP included in the guidelines and consensus documents?<br>Likert scale: 0 not at all improvable→ 5 very improvable
**Item 9: Cost associated with the management of a patient with CKD-aP**	Question: How do you consider the cost associated with the management of a patient with CKD-aP compared to one without pruritus?<br>Likert scale: 0 low → 5 high

Part 3. Scenario for ideal CKD-aP treatment by BWS

Scenario 1

**Attributes**	**Best**	**Worst**
The treatment is administered in the dialysis circuit after each dialysis session	☐	☐
Treatment reduces itch with statistical significance	☐	☐
The treatment does not interact with other drugs commonly used in HD	☐	☐
The treatment does not cause withdrawal/dependence syndrome	☐	☐

Scenario 2

CONFIDENCIAL

Attributes	Best	Worst
The treatment does not cause withdrawal/dependence syndrome	☐	☐
Treatment improves overall QoL (physical and mental)	☐	☐
Treatment improves sleep quality	☐	☐
Treatment is supported by clinical development/significant evidence and has Spanish Agency of Medicines and Medical Devices approved indication for pruritus	☐	☐
**Scenario 3**		
**Attributes**	**Best**	**Worst**
The treatment is administered in the dialysis circuit after each dialysis session	☐	☐
The treatment does not interact with other drugs commonly used in HD	☐	☐
Treatment improves overall QoL (physical and mental)	☐	☐
Treatment is supported by clinical development/significant evidence and has Spanish Agency of Medicines and Medical Devices approved indication for pruritus	☐	☐
**Scenario 4**		
**Attributes**	**Best**	**Worst**
The treatment does not interact with other drugs commonly used in HD	☐	☐
The treatment does not cause withdrawal/dependence syndrome	☐	☐
Treatment improves sleep quality	☐	☐
Treatment is supported by clinical development/significant evidence and has Spanish Agency of Medicines and Medical Devices approved indication for pruritus	☐	☐
**Scenario 5**		
**Attributes**	**Best**	**Worst**
The treatment is administered in the dialysis circuit after each dialysis session	☐	☐
Treatment reduces itch with statistical significance	☐	☐
Treatment improves sleep quality	☐	☐
Treatment is supported by clinical development/significant evidence and has Spanish Agency of Medicines and Medical Devices approved indication for pruritus	☐	☐
**Scenario 6**		
**Attributes**	**Best**	**Worst**
Treatment reduces itch with statistical significance	☐	☐

CONFIDENCIAL

The treatment does not interact with other drugs commonly used in HD	☐	☐
Treatment improves overall QoL (physical and mental)	☐	☐
Treatment improves sleep quality	☐	☐
**Scenario 7**		
**Attributes**	**Best**	**Worst**
The treatment is effective after a maximum of 2 weeks from the start of treatment	☐	☐
The treatment does not interact with other drugs commonly used in HD	☐	☐
Treatment improves overall QoL (physical and mental)	☐	☐
Treatment improves sleep quality	☐	☐
**Scenario 8**		
**Attributes**	**Best**	**Worst**
The treatment does not interact with other drugs commonly used in HD	☐	☐
Treatment reduces itch with statistical significance	☐	☐
Treatment improves overall QoL (physical and mental)	☐	☐
Treatment improves sleep quality	☐	☐
**Scenario 9**		
**Attributes**	**Best**	**Worst**
The treatment is effective after a maximum of 2 weeks from the start of treatment	☐	☐
Treatment reduces itch with statistical significance	☐	☐
Treatment improves overall QoL (physical and mental)	☐	☐
Treatment is supported by clinical development/significant evidence and has Spanish Agency of Medicines and Medical Devices approved indication for pruritus	☐	☐
**Scenario 10**		
**Attributes**	**Best**	**Worst**
The treatment is administered in the dialysis circuit after each dialysis session	☐	☐
The treatment is effective after a maximum of 2 weeks from the start of treatment	☐	☐
Treatment improves overall QoL (physical and mental)	☐	☐
The treatment does not cause withdrawal/dependence syndrome	☐	☐

CONFIDENCIAL

Scenario 11		
**Attributes**	**Best**	**Worst**
The treatment is effective after a maximum of 2 weeks from the start of treatment	☐	☐
Treatment reduces itch with statistical significance	☐	☐
The treatment does not cause withdrawal/dependence syndrome	☐	☐
Treatment improves sleep quality	☐	☐
**Scenario 12**		
**Attributes**	**Best**	**Worst**
The treatment is administered in the dialysis circuit after each dialysis session	☐	☐
The treatment is effective after a maximum of 2 weeks from the start of treatment	☐	☐
The treatment does not cause withdrawal/dependence syndrome	☐	☐
Treatment is supported by clinical development/significant evidence and has Spanish Agency of Medicines and Medical Devices approved indication for pruritus	☐	☐
**Scenario 13**		
**Attributes**	**Best**	**Worst**
The treatment is effective after a maximum of 2 weeks from the start of treatment	☐	☐
Treatment reduces itch with statistical significance	☐	☐
The treatment does not interact with other drugs commonly used in HD	☐	☐
Treatment is supported by clinical development/significant evidence and has Spanish Agency of Medicines and Medical Devices approved indication for pruritus	☐	☐
**Scenario 14**		
**Attributes**	**Best**	**Worst**
The treatment is administered in the dialysis circuit after each dialysis session	☐	☐
The treatment is effective after a maximum of 2 weeks from the start of treatment	☐	☐
The treatment does not interact with other drugs commonly used in HD	☐	☐
Treatment improves sleep quality	☐	☐

HD: haemodialysis. QoL: quality of life.

CONFIDENTIAL

1.3. Table S3. Results. Sociodemographic characteristics

Participants' sociodemographic characteristics	
Age:<br>years, mean (SD)	53,3 (6,9)
Sex:<br>Male, n (%)<br>Female, n (%)	 12 (57,1)<br>9 (42,9)
CC.AA.:	
Andalucía, n (%)	1 (4,8)
Aragón, n (%)	0 (0,0)
Asturias, n (%)	0 (0,0)
Baleares, n (%)	1 (4,8)
Canarias, n (%)	0 (0,0)
Cantabria, n (%)	0 (0,0)
Castilla- La Mancha, n (%)	1 (4,8)
Castilla y León, n (%)	2 (9,5)
Cataluña, n (%)	0 (0,0)
Comunidad Valenciana, n (%)	1 (4,8)
Extremadura, n (%)	1 (4,8)
Galicia, n (%)	4 (19,0)
La Rioja, n (%)	0 (0,0)
Madrid, n (%)	5 (23,8)
Murcia, n (%)	1 (4,8)
Navarra, n (%)	1 (4,8)
País Vasco, n (%)	3 (14,3)
Ceuta y Melilla, n (%)	0 (0,0)
Profile:	
Nephrologists, n (%)	5 (23,8)
Hospital pharmacists, n (%)	5 (23,8)
Nurses, n (%)	3 (14,3)
Pacient representative, n (%)	4 (19,0)
Regional health authorities, n (%)	4 (19,0)
**Sociodemographic characteristics by profile**	

Profile	Nephrologists (n=5)	Hospital pharmacists (n=5)	Nurses (n=3)	Pacient representative (n=4)	Regional health authorities (n=4)
Age:<br>Years, mean (SD)	56,0 (6,9)	51,3 (3,2)	53,0 (4,8)	53,3 (10,5)	51,8 (9,3)
Sex:<br>Male, n (%)<br>Female, n (%)	 3 (60,0)<br>2 (40,0)	 3 (60,0)<br>2 (40,0)	 1 (33,3)<br>2 (66,7)	 3 (75,0)<br>1 (25,0)	 2 (50,0)<br>2 (50,0)
CC.AA.:					
Andalucía, n (%)	0 (0,0)	0 (0,0)	0 (0,0)	0 (0,0)	0 (0,0)
Aragón, n (%)	0 (0,0)	0 (0,0)	0 (0,0)	0 (0,0)	0 (0,0)
Asturias, n (%)	0 (0,0)	0 (0,0)	0 (0,0)	0 (0,0)	0 (0,0)
Baleares, n (%)	0 (0,0)	1 (20,0)	0 (0,0)	0 (0,0)	0 (0,0)
Canarias, n (%)	0 (0,0)	0 (0,0)	0 (0,0)	0 (0,0)	0 (0,0)
Cantabria, n (%)	0 (0,0)	0 (0,0)	0 (0,0)	0 (0,0)	0 (0,0)
Castilla- La Mancha, n (%)	0 (0,0)	0 (0,0)	0 (0,0)	1 (20,0)	0 (0,0)
Castilla y León, n (%)	0 (0,0)	0 (0,0)	0 (0,0)	1 (20,0)	1 (20,0)
Cataluña, n (%)	0 (0,0)	0 (0,0)	0 (0,0)	0 (0,0)	0 (0,0)
Comunidad Valenciana, n (%)	0 (0,0)	0 (0,0)	0 (0,0)	0 (0,0)	1 (20,0)
Extremadura, n (%)	1 (20,0)	0 (0,0)	0 (0,0)	0 (0,0)	0 (0,0)
Galicia, n (%)	1 (20,0)	2 (40,0)	0 (0,0)	0 (0,0)	0 (0,0)
La Rioja, n (%)	0 (0,0)	0 (0,0)	0 (0,0)	0 (0,0)	0 (0,0)
Madrid, n (%)	1 (20,0)	1 (20,0)	2 (40,0)	1 (20,0)	0 (0,0)
Murcia, n (%)	1 (20,0)	0 (0,0)	0 (0,0)	0 (0,0)	0 (0,0)
Navarra, n (%)	0 (0,0)	0 (0,0)	1 (20,0)	0 (0,0)	0 (0,0)
País Vasco, n (%)	1 (20,0)	1 (20,0)	0 (0,0)	0 (0,0)	1 (20,0)
Ceuta y Melilla, n (%)	0 (0,0)	0 (0,0)	0 (0,0)	0 (0,0)	0 (0,0)

SD: Standar deviation. Measures the amount of variability, or dispersion, from the individual data values to the mean.

1.4. Table S4. Unmet needs identified by profile

Items about CKD-aP pathology management	Mean (SD)	Min	Max
**Nephrologist (n=5)**			
Question: In your opinion, how severe is CKD-aP?	4,6 (0,5)	4,0	5,0

Likert scale: 0 not at all severe → 5 very severe			
Question: In your opinion, how much does CKD-aP affect patient QoL?<br>Likert scale: 0 not at all → 5 a lot	4,0 (1,0)	3,0	5,0
Question: In your opinion, how much of the population is affected by CKD-aP?<br>Likert scale: 0 low affected population→ 5 high affected population	3,4 (0,9)	3,0	5,0
Question: Regarding the total number of patients suffering from CKD-aP, in your opinion, how is the CKD-aP rate of diagnosis?<br>Likert scale: 0 low → 5 high	1,6 (0,9)	1,0	3,0
Question: In your opinion, how is the level of knowledge about CKD-aP in agents involved in the pathology?<br>Likert scale: 0 low → 5 high	2,2 (0,4)	2,0	3,0
Question: Although there are no authorised drugs available in Spain for CKD-aP, in your opinion, how effective are the treatments currently used?<br>Likert scale: 0 not at all effective → 5 very effective	2,4 (0,5)	2,0	3,0
Question: Although there are no authorised drugs available in Spain for CKD-aP, in your opinion, how safe are the treatments currently used?<br>Likert scale: 0 not at all safe → 5 very safe	2,4 (0,9)	2,0	4,0
Question: In your opinion, how are the current patterns for CKD-aP management which are included in the guidelines and consensus documents?<br>Likert scale: 0 no improvement needed → 5 a lot of improvement needed	2,4 (1,3)	1,0	4,0
Question: In your opinion, what is the cost associated with the management of a patient with CKD-aP compared to one without pruritus?<br>Likert scale: 0 low → 5 high	3,8 (0,8)	3,0	5,0
**Hospital pharmacists (n=5)**			
Question: In your opinion, how severe is CKD-aP?<br>Likert scale: 0 not at all severe → 5 very severe	3,4 (0,5)	3,0	4,0
Question: In your opinion, how much does CKD-aP affect patient QoL?<br>Likert scale: 0 not at all → 5 a lot	4,2 (0,4)	4,0	5,0

CONFIDENCIAL

Question: In your opinion, how much of the population is affected by CKD-aP?<br>Likert scale: 0 low affected population→ 5 high affected population	3,2 (1,3)	2,0	5,0
Question: Regarding the total number of patients suffering from CKD-aP, in your opinion, how is the CKD-aP rate of diagnosis?<br>Likert scale: 0 low → 5 high	1,8 (0,8)	1,0	3,0
Question: In your opinion, how is the level of knowledge about CKD-aP in agents involved in the pathology?<br>Likert scale: 0 low → 5 high	2,0 (1,2)	1,0	4,0
Question: Although there are no authorised drugs available in Spain for CKD-aP, in your opinion, how effective are the treatments currently used?<br>Likert scale: 0 not at all effective → 5 very effective	1,8 (0,4)	1,0	2,0
Question: Although there are no authorised drugs available in Spain for CKD-aP, in your opinion, how safe are the treatments currently used?<br>Likert scale: 0 not at all safe → 5 very safe	3,0 (0,7)	2,0	4,0
Question: In your opinion, how are the current patterns for CKD-aP management which are included in the guidelines and consensus documents?<br>Likert scale: 0 no improvement needed → 5 a lot of improvement needed	3,8 (0,8)	3,0	5,0
Question: In your opinion, what is the cost associated with the management of a patient with CKD-aP compared to one without pruritus?<br>Likert scale: 0 low → 5 high	3,2 (0,8)	3,0	5,0
**Nurses (n=3)**			
Question: In your opinion, how severe is CKD-aP?<br>Likert scale: 0 not at all severe → 5 very severe	3,7 (0,6)	3,0	4,0
Question: In your opinion, how much does CKD-aP affect patient QoL?<br>Likert scale: 0 not at all → 5 a lot	5,0 (0,0)	5,0	5,0
Question: In your opinion, how much of the population is affected by CKD-aP?<br>Likert scale: 0 low affected population→ 5 high affected population	4,3 (0,6)	4,0	5,0

CONFIDENCIAL

Question: Regarding the total number of patients suffering from CKD-aP, in your opinion, how is the CKD-aP rate of diagnosis?<br>Likert scale: 0 low → 5 high	0,7 (0,6)	0,0	1,0
Question: In your opinion, how is the level of knowledge about CKD-aP in agents involved in the pathology?<br>Likert scale: 0 low → 5 high	2,0 (1,0)	1,0	3,0
Question: Although there are no authorised drugs available in Spain for CKD-aP, in your opinion, how effective are the treatments currently used?<br>Likert scale: 0 not at all effective → 5 very effective	1,3 (0,6)	1,0	2,0
Question: Although there are no authorised drugs available in Spain for CKD-aP, in your opinion, how safe are the treatments currently used?<br>Likert scale: 0 not at all safe → 5 very safe	2,0 (0,0)	2,0	2,0
Question: In your opinion, how are the current patterns for CKD-aP management which are included in the guidelines and consensus documents?<br>Likert scale: 0 no improvement needed → 5 a lot of improvement needed	2,3 (2,1)	0,0	4,0
Question: In your opinion, what is the cost associated with the management of a patient with CKD-aP compared to one without pruritus?<br>Likert scale: 0 low → 5 high	4,0 (1,0)	3,0	5,0
**Patient representative (n=4)**			
Question: In your opinion, how severe is CKD-aP?<br>Likert scale: 0 not at all severe → 5 very severe	3,7 (0,5)	3,0	4,0
Question: In your opinion, how much does CKD-aP affect patient QoL?<br>Likert scale: 0 not at all → 5 a lot	4,0 (0,0)	4,0	4,0
Question: In your opinion, how much of the population is affected by CKD-aP?<br>Likert scale: 0 low affected population→ 5 high affected population	3,2 (1,3)	2,0	5,0
Question: Regarding the total number of patients suffering from CKD-aP, in your opinion, how is the CKD-aP rate of diagnosis?<br>Likert scale: 0 low → 5 high	2,0 (1,2)	1,0	3,0
Question: In your opinion, how is the level of knowledge about CKD-aP in agents involved in the pathology?	1,7 (0,5)	1,0	2,0

CONFIDENCIAL

Likert scale: 0 low → 5 high			
Question: Although there are no authorised drugs available in Spain for CKD-aP, in your opinion, how effective are the treatments currently used?<br>Likert scale: 0 not at all effective → 5 very effective	1,5 (0,6)	1,0	2,0
Question: Although there are no authorised drugs available in Spain for CKD-aP, in your opinion, how safe are the treatments currently used?<br>Likert scale: 0 not at all safe → 5 very safe	2,5 (0,6)	2,0	3,0
Question: In your opinion, how are the current patterns for CKD-aP management which are included in the guidelines and consensus documents?<br>Likert scale: 0 no improvement needed → 5 a lot of improvement needed	3,0 (0,8)	2,0	4,0
Question: In your opinion, what is the cost associated with the management of a patient with CKD-aP compared to one without pruritus?<br>Likert scale: 0 low → 5 high	3,5 (1,0)	2,0	4,0
**Regional health authorities (n=4)**			
Question: In your opinion, how severe is CKD-aP?<br>Likert scale: 0 not at all severe → 5 very severe	3,7 (0,5)	3,0	4,0
Question: In your opinion, how much does CKD-aP affect patient QoL?<br>Likert scale: 0 not at all → 5 a lot	3,7 (0,5)	3,0	4,0
Question: In your opinion, how much of the population is affected by CKD-aP?<br>Likert scale: 0 low affected population→ 5 high affected population	2,2 (0,5)	2,0	3,0
Question: Regarding the total number of patients suffering from CKD-aP, in your opinion, how is the CKD-aP rate of diagnosis?<br>Likert scale: 0 low → 5 high	2,2 (1,3)	1,0	4,0
Question: In your opinion, how is the level of knowledge about CKD-aP in agents involved in the pathology?<br>Likert scale: 0 low → 5 high	3,0 (1,2)	2,0	4,0
Question: Although there are no authorised drugs available in Spain for CKD-aP, in your opinion, how effective are the treatments currently used?<br>Likert scale: 0 not at all effective → 5 very effective	2,2 (1,0)	1,0	3,0

CONFIDENCIAL

Question: Although there are no authorised drugs available in Spain for CKD-aP, in your opinion, how safe are the treatments currently used?<br><br>Likert scale: 0 not at all safe → 5 very safe	3,2 (1,5)	2,0	5,0
Question: In your opinion, how are the current patterns for CKD-aP management which are included in the guidelines and consensus documents?<br><br>Likert scale: 0 no improvement needed → 5 a lot of improvement needed	3,2 (0,5)	3,0	4,0
Question: In your opinion, what is the cost associated with the management of a patient with CKD-aP compared to one without pruritus?<br><br>Likert scale: 0 low → 5 high	2,5 (1,0)	1,0	3,0

SD: Standar deviation. Measures the amount of variability, or dispersion, from the individual data values to the mean. Min: is the smallest value in the data set given by participants. Max: is the largest value in the data set given by participants.

1.5. Table S5. Unmet needs. Variability in responses

n=21	Mean	SD	Min	Max
Question: In your opinion, how severe is CKD-aP?<br><br>Likert scale: 0 not at all severe → 5 very severe	3.71	0.64	3.0	5.0
Question: In your opinion, how much does CKD-aP affect patient QoL?<br><br>Likert scale: 0 not at all → 5 a lot	4.29	0.56	3.0	5.0
Question: In your opinion, how much of the population is affected by CKD-aP?<br><br>Likert scale: 0 low affected population→ 5 high affected population	3.24	1.09	2.0	5.0
Question: Regarding the total number of patients suffering from CKD-aP, in your opinion, how is the CKD-aP rate of diagnosis?<br><br>Likert scale: 0 low → 5 high	1.71	1.01	0.0	4.0
Question: In your opinion, how is the level of knowledge about CKD-aP in agents involved in the pathology?<br><br>Likert scale: 0 low → 5 high	2.19	0.93	1.0	4.0
Question: Although there are no authorised drugs available in Spain for CKD-aP, in your opinion, how effective are the treatments currently used?	1.91	0.70	1.0	3.0

Confidencial No está permitida su distribución

Likert scale: 0 not at all effective → 5 very effective				
Question: Although there are no authorised drugs available in Spain for CKD-aP, in your opinion, how safe are the treatments currently used?<br>Likert scale: 0 not at all safe → 5 very safe	2.67	0.91	2.0	5.0
Question: In your opinion, how are the current patterns for CKD-aP management which are included in the guidelines and consensus documents?<br>Likert scale: 0 no improvement needed → 5 a lot of improvement needed	3.00	1.18	0.0	5.0
Question: In your opinion, what is the cost associated with the management of a patient with CKD-aP compared to one without pruritus?<br>Likert scale: 0 low → 5 high	3.38	0.97	1.0	5.0

SD: Standar deviation. Measures the amount of variability, or dispersion, from the individual data values to the mean. Min: is the smallest value in the data set given by participants. Max: is the largest value in the data set given by participants.

1.6. Table S6. Preferences about which attributes the ideal CKD-aP treatment should have, detailed per group.

Attributes	Best	Worst	B-W score
**Nephrologist (n=5)**			
Treatment improves overall QoL (physical and mental)	21,0	0,0	21,0
Treatment reduces itch with statistical significance	20,0	0,0	20,0
Treatment is supported by clinical development/significant evidence and has Spanish Agency of Medicines and Medical Devices approved indication for pruritus	21,0	6,0	15,0
Treatment improves sleep quality	4,0	1,0	3,0
The treatment does not interact with other drugs commonly used in HD	2,0	10,0	-8,0
The treatment does not cause withdrawal/dependence syndrome	2,0	13,0	-11,0

CONFIDENCIAL

The treatment is effective after a maximum of 2 weeks from the start of treatment	0,0	15,0	-15,0
The treatment is administered in the dialysis circuit after each dialysis session	0,0	25,0	-25,0
**Hospital pharmacists (n=5)**			
Treatment improves overall QoL (physical and mental)	16,0	3,0	13,0
Treatment reduces itch with statistical significance	19,0	2,0	17,0
Treatment is supported by clinical development/significant evidence and has Spanish Agency of Medicines and Medical Devices approved indication for pruritus	24,0	1,0	23,0
Treatment improves sleep quality	8,0	6,0	2,0
The treatment does not interact with other drugs commonly used in HD	1,0	6,0	-5,0
The treatment does not cause withdrawal/dependence syndrome	2,0	6,0	-4,0
The treatment is effective after a maximum of 2 weeks from the start of treatment	0,0	21,0	-21,0
The treatment is administered in the dialysis circuit after each dialysis session	0,0	25,0	-25,0
**Nurses (n=3)**			
Treatment improves overall QoL (physical and mental)	15,0	0,0	15,0
Treatment reduces itch with statistical significance	10,0	0,0	10,0
Treatment is supported by clinical development/significant evidence and has Spanish Agency of Medicines and Medical Devices approved indication for pruritus	9,0	1,0	8,0
Treatment improves sleep quality	3,0	0,0	3,0
The treatment does not interact with other drugs commonly used in HD	5,0	5,0	0,0
The treatment does not cause withdrawal/dependence syndrome	0,0	8,0	-8,0
The treatment is effective after a maximum of 2 weeks from the start of treatment	0,0	15,0	-15,0
The treatment is administered in the dialysis circuit after each dialysis session	0,0	13,0	-13,0

CONFIDENTIAL

Patient representative (n=4)			
Treatment improves overall QoL (physical and mental)	18,0	0,0	18,0
Treatment reduces itch with statistical significance	8,0	0,0	8,0
Treatment is supported by clinical development/significant evidence and has Spanish Agency of Medicines and Medical Devices approved indication for pruritus	11,0	3,0	8,0
Treatment improves sleep quality	11,0	7,0	4,0
The treatment does not interact with other drugs commonly used in HD	3,0	3,0	0,0
The treatment does not cause withdrawal/dependence syndrome	3,0	16,0	-13,0
The treatment is effective after a maximum of 2 weeks from the start of treatment	1,0	13,0	-12,0
The treatment is administered in the dialysis circuit after each dialysis session	1,0	14,0	-13,0
**Regional health authorities (n=4)**			
Treatment improves overall QoL (physical and mental)	16,0	2,0	14,0
Treatment reduces itch with statistical significance	14,0	0,0	14,0
Treatment is supported by clinical development/significant evidence and has Spanish Agency of Medicines and Medical Devices approved indication for pruritus	13,0	2,0	11,0
Treatment improves sleep quality	6,0	4,0	2,0
The treatment does not interact with other drugs commonly used in HD	3,0	1,0	2,0
The treatment does not cause withdrawal/dependence syndrome	2,0	14,0	-12,0
The treatment is effective after a maximum of 2 weeks from the start of treatment	2,0	11,0	-9,0
The treatment is administered in the dialysis circuit after each dialysis session	0,0	22,0	-22,0

Higher values of B-W score indicate that the attribute was chosen more frequently as most preferred, and negative values indicate that the attribute was chosen more frequently as least preferred.

CONFIDENCIAL
